# The role of stereotypical information on medical judgements for black and white patients

**DOI:** 10.1371/journal.pone.0268888

**Published:** 2022-06-08

**Authors:** Filipa Madeira, Rui Costa-Lopes, Emerson Araújo Do Bú, Rui Tato Marinho

**Affiliations:** 1 Institute of Social Sciences, University of Lisbon, Lisbon, Portugal; 2 Faculty of Psychology, University of Lisbon, Lisbon, Portugal; 3 Gastroenterology and Hepatology Department, Centro Hospitalar Universitário de Lisboa Norte, Medical School of Lisbon, National Program for Viral Hepatitis, Ministry of Health, Lisbon, Portugal; University of Granada: Universidad de Granada, SPAIN

## Abstract

THIS ARTICLE USES WORDS OR LANGUAGE THAT IS CONSIDERED PROFANE, VULGAR, OR OFFENSIVE BY SOME READERS. The new generation of direct-acting antivirals has improved dramatically the rates of cure for chronic hepatitis C. Yet, evidence shows that racial groups are deemed more often ineligible for hepatitis C treatment, despite no clinical evidence supporting differential treatment for Black and White patients. One possible explanation has to do with providers’ racial biases. This investigation sought to explore medical students’ racial stereotypes (Study 1, *N* = 171) and the role of stereotypical cues on perceptions of medical adherence of Black and White patients (Study 2, *N* = 208). In Study 1, we first sought to identify health-related aspects that are consistently associated with Blacks as part of a stereotype. In Study 2, we experimentally manipulated racial stereotypes identified in Study 1 by asking participants to read a clinical vignette depicting a patient (Black *vs*. White) and their medical history (cause of exposure to hepatitis C: unprotected sex *vs*. non-injectable drugs use). The results show that the impact of stereotypicality on patient perceived compliance varies as a function of medical students’ racial prejudice. Implications for further applied health inequalities research and for medical training are discussed.

## Introduction

Disclaimer: This article uses words or language that is considered profane, vulgar or offensive by some readers. Due to the topic studied in this article, quoting offensive language is academically justified but we nor PLOS in no way endorse the use of these words or the content of the quotes. Likewise, the quotes do not represent the opinions of us or that of PLOS, and we condemn online harassment and offensive language. Medical providers are responsible for making daily clinical decisions about patients. Some of the resulting resolutions have a low impact on patients’ lives, but others have a deeper impact on them, compromising the quality, or even the preservation, of life [1]. In this paper, we focus on socially critical decisions—a form of social behaviors with a deep impact on people’s lives—particularly in scenarios where existing resources are scarce or limited, and for this reason, may imply an unequal allocation of material resources [2]. Hepatitis C treatment is a paradigmatic example of a medical scenario where medical decisions have a great impact on people’s lives, involving the allocation of limited or heavily burdened resources to the National Health System. Hepatitis C virus (HCV) infection is a major public health problem worldwide due to the high rate of progression to chronic and the potential for progression to cirrhosis, liver cancer and death [3]. Yet, the new generation of direct-acting antivirals, which are medications targeted at specific steps within the Hepatitis C virus (HCV), has improved the rates of cure dramatically, i.e., 97% of permanent viral eradication [4]. As a result, hepatitis C has gone from being an incurable infectious disease to being a treatable and, importantly, curable disease. Notably, this has led the World Health Organization to set the objective of eradicating hepatitis C by 2030 [5]. This is to be achieved by ensuring an equitable access to recommended testing, care and treatment services for all [3].

Guided by these goals, Portugal implemented a structural policy to eradicate the disease as a public health threat. In 2015, the National Authority of Medicines and Health Products, I.P. [6] approved direct-acting antiviral drugs for prescription. As a result, all people who are eligible for treatment and diagnosed with HCV infections have access to oral direct-acting antivirals (DAAs) treatment. However, the high cost of AADs represents not only a challenge to the sustainability of the National Health Service, but also an obstacle to achieving the goals of eradicating the hepatitis C virus. One of the effects already felt are that some patients are waiting a long time accessing HCV treatments [7].

Another potential consequence is the discretionary power of decision associated with rationalizing HCV treatments. It is possible that patient prioritization criteria will increase the likelihood of racially discriminatory recommendation practices occurring. If a patient is part of a group about whom there is the stereotype of low health competence or high risk behaviors, it is possible that this stereotypical inference may unconsciously influence medical decision making in patient prioritization [8], by considering a patient of a racial group as a potentially poor candidate for an expensive treatment. The current work explores medical students’ health-related stereotypes about Black African patients, and experimentally tests whether stereotypical information influences the judgments that medical students form about the therapeutic adherence of Black and White patients.

### Racial biases in HCV treatment

Even though there is no clinical evidence indicating that Black patients should be more often deemed ineligible than other racial groups for HCV treatment, the medical literature has provided initial evidence of racial disparities in HCV treatment eligibility. Accordingly, Black patients are less likely to receive antiviral treatment for chronic hepatitis C even after adjusting for socioeconomic status and medical/psychiatric comorbidities [4,9,10], particularly in the US context. These findings suggest that one possible underlying contributor to the HCV treatment eligibility disparity disfavoring Black patients could be the existence of racial biases at the level of the provider.

### Provider bias and healthcare inequalities

Biases are formed by systems of a cognitive and affective nature, and are generally defined as a tendency to evaluate in-group members more favorably than out-group members [11]. Provider biases are often activated without awareness, when interacting with others race or ethnicity, and may assume the form of stereotyping, or be expressed in the form of prejudice. A good amount of research has shown among various medical domains that the salience of a patient’s racial category is sufficient to trigger provider biases, negatively affecting interpersonal communication and medical decisions [12–15]. Research has consistently found that provider bias affects their interactions with racial minority patients, particularly Black patients [16–20]. For example, Penner et al. [18] examined implicit racial bias in relation to interpersonal behavior, showing that more biased providers were rated by their African-American patients as lower in warmth and friendliness. Moreover, a provider with high implicit racial bias tends to be less patient-centered and spend less time providing treatment planning, health education and answering questions of Black patients (*vs*. White) [16,19,20].

Regarding clinical decisions, evidence is mixed about whether these are affected by provider bias, with studies failing to find effects of prejudice on provider’s treatment decisions [21–24]. However, the evidence does show that bias influences, at least, some types of clinical decisions, particularly bias at the implicit level [25,26]. For example, Green and collaborators [25] found that providers with greater implicit racial bias were less likely to recommend the appropriate treatment when presented with a clinical vignette portraying a patient with chest pain symptoms. In another study on treatment decisions, Sabin and Greenwald [26] found that the higher the provider level of bias was, the less likely they were to prescribe postsurgical pain medication for a Black (*vs*. White) patient.

Another type of bias, important in a scenario of medical resource allocation, concerns providers’ perceptions of patient competence in health. van Ryn et al. [27] investigated physicians’ beliefs about Black and White patients who were appropriate candidates for coronary bypass surgery. Physicians were asked to rate each of their patients (both Black and White) in characteristics such as education and intelligence and in dimensions of health competence such as compliance with medical advice and participation in cardiac rehabilitation (if prescribed). Black patients were rated as less educated and intelligent than White patients, and importantly, were perceived as more likely to fail to comply with medical advice and less likely to participate in cardiac rehabilitation than their White counterparts. Importantly, Black men were also less recommended for bypass surgery than were White men, and this was partially explained by physicians’ perceptions of patients, in that the less educated Black patients were perceived to be, the less they were recommended for medical surgery. Thus, this suggests the possibility of Black patients being indirectly perceived as poorer candidates for the medical procedure. Following this reasoning, providers’ beliefs about Black patients may influence their behavior and decision-making. If the physicians’ perception of a patient’s medical adherence is influenced by their social category, there is a possibility that this association will negatively affect the way a candidate for treatment is evaluated. Especially if this candidate is a Black person.

### Racial stereotypes and health inequalities

Physicians’ perceptions behind potentially discriminatory treatment decisions are often grounded on stereotypes. Stereotypes are beliefs about traits more associated with certain social groups [28] and, as such, include expectations about the occurrence of a certain trait when thinking about a member of a specific social group. Providers often hold both explicit and implicit racial stereotypes [29–31]. At the explicit level, van Ryn and colleagues surveyed physicians’ perceptions of Black and White patients and found that, relative to White patients, Black patients are perceived as more likely to be seen as at risk for substance abuse, and as having inadequate social support and less likely to adhere to medical advice [30]. At the implicit level, findings suggest that providers associate Blacks with certain stereotypes, such as non-compliance and reduced cooperativeness. For example, being Black was more strongly associated with being less intelligent, less cooperative, and less compliant than White patients [25,26,32,33]. Besides the traits mentioned, being Black is also more quickly associated with certain medical conditions. Moskowitz et al. [34] found, for example, that priming an African American face led providers to react more quickly for stereotypical diseases, with a known genetic component (e.g., sickle cell anemia), but importantly, also to others without genetic association, such as obesity or drug abuse.

Moreover, the racial stereotypes that providers consciously and unconsciously hold about Blacks are also likely to influence clinical and juridical judgments or may even unduly influence treatment recommendations [29,31,34,35]. For instance, Calabrese and colleagues [35] examined medical students’ willingness to prescribe antiretroviral pre-exposure prophylaxis (PrEP) to a White or a Back patient. Medical students were asked, among other questions, about the likelihood of the patient engaging in more unprotected sex if prescribed PrEP, patient’s level of adherence to PrEP and about PrEP prescription. Overall, medical students were equally likely to prescribe PrEP for the Black and White patients. However, relative to the White, the Black patient was perceived as more likely to increase his rate of unprotected sex if prescribed PrEP, and this belief decreased medical students’ willingness to prescribe PrEP for the Black patient. In other words, because of race-based sexual stereotypical inferences, medical students determined that Black patients were poor candidates for the prophylactic medication.

These findings show that inferences driven from group-based stereotypes account for some of the racial differences in the medical recommendation for PrEP. We suggest that providers’ vulnerability to stereotyping occurs in other medical domains, particularly in scenarios where existing resources are scarce or limited, and for this reason, may impact greatly on people’s lives.

Stereotypes can have a subtle yet biasing influence on the way people perceive, form impressions and make judgments of others [36]. Thus, it is plausible to assume that when a stereotype cue is paired with social categorization processes, it triggers the negative component of the stereotype, which is likely to influence how providers evaluate candidates for medical treatment. If providers are more likely to associate Blacks as non-compliant patients [27], then this effect is more likely to occur in a situation where the provider faces a situation with a Black patient displaying a behavior congruent with stereotypes associated with Black people (though the stereotypical behavior is not related with the issue of compliance). Moreover, the impact of stereotyping on the perception of patient compliance can be enhanced by providers’ racial prejudice. Provider’s level of racial bias was shown to interact with patient race, demonstrating that Black patients were less likely to be recommended for medical treatment, especially among physicians high in implicit prejudice [25]. Thus, the current work goes further suggesting that the impact of a group stereotype inferred from a stereotypic cue is likely to result in less favorable compliance ratings for Black patients, particularly among providers where racial bias is high.

Literature on stereotype activation contends that when providing information stereotypically consistent with characteristics of Blacks as a group, outcomes may be more likely to be unfavorable toward the targeted person [37,38], compared to stereotypically inconsistent information. In the study of Bodenhausen and Wyer [38], after reading a description of a job-related infraction committed by the employee, participants were asked to give their recommendation for how severe the target should be disciplined, and how severe should the punishment be if the problem occurred again. The target was either American or Arab and the offence was *stereotypically* American or Arab. Overall, Arab targets were treated more severely for infractions than were American targets. Importantly, more severe punishment was found for stereotypic offences than for non-stereotypic ones. Thus, we reason that when the Black patient appears associated with a stereotypic cue (i.e. sexual risk behavior), a less favorable evaluation is more likely to occur. In contrast, when the Black patient appears associated with a non-stereotypic cue, he will be more likely to be positively evaluated.

Although some research on medical decision-making has focused on the role of explicit racial stereotypes, to our knowledge, it is not yet known how the salience of stereotypical information affects judgments of medical adherence (and treatment decisions) for Black and White patients with hepatitis C. And, more importantly, to what extent is the use of such stereotypic information influenced by the medical student’s level of racial prejudice to disadvantage Black patients. Thus, while attempting to contribute to the literature demonstrating the influence of stereotypes on perceptions of compliance and treatment decisions, this research seeks to further advance it with a unique contribution based on the fact that it a) experimentally manipulates stereotypicality in the context of medical judgment by providing information on behavior about patients not directly associated with compliance (thus eliminating endogenous explanations), b) analyses how the influence of those stereotypes may depend on the providers’ level of racial prejudice, all the while focusing on a different disease and treatment, and conducted in a different national context (outside the US and in a country with a full public state-subsidized health care system).

In the current work, we first surveyed medical students exploring the content of stereotypes associated with Black Africans in the health care context (Study 1). Then, based on the content of stereotypic representations, we investigated the relationship between patient race, stereotype congruency and perceptions about HCV-treatment patient compliance among medical students (Study 2). To this end, we created two medical scenarios—(a) a stereotypically-black clinical case and (b) a non-stereotypically black clinical case–and examined to what extent the relationship between patient race and HCV-treatment evaluation changed as a function of the stereotypic information and the level of racial prejudice. We hypothesized that the Black patient is likely to be perceived as less compliant in medical cases involving stereotypic behaviors, than in cases involving non-stereotypic ones. In addition, this effect is thought to be stronger among individuals high in racial prejudice. Considering that past research has shown that the effect of stereotypes on medical judgments is more likely to occur for the dimension of compliance [35] than in the treatment decision in itself, and that the treatment decision is more likely to be undermined by social desirability issues, we developed no hypotheses for the medical recommendation in itself. Datasets used in this research program can be accessed at the OSF repository platform: https://osf.io/7eh8r

## Study 1. Stereotypes associated with Black Africans in the healthcare context

Using qualitative methods, we first sought to identify the content of racial stereotypes that could serve as content for developing material to be included as stereotypical cues portrayed in Study 2. Thus, to examine what health-related aspects are consistently associated with Blacks as part of a stereotype, we first surveyed medical students to explore the content of behaviors associated with Black Africans in the health care context.

### Method

#### Participants

One hundred and seventy-one White Portuguese medical students were invited to take part in a research study on medical decision-making processes. One hundred and fifty-three ranging from first to sixth medical school year (*M* = 3.86, *SD* = 1.30) responded to an open question about cultural stereotypes about health behaviors of Black Africans, presented at the end of the survey; their ages ranged from 18 to 31 years (68% female, Age: *M* = 21.56, *SD* = 2.09). Ethical approval was obtained from the Ethics Committee of the Faculty of Medicine of the University of Lisbon.

#### Procedure

Based on the paradigm developed by Devine [36] and with the aim of capturing health-related behaviors commonly associated with Black Africans by health professionals, participants were asked to read the following introduction: “we are all aware of the existence of cultural stereotypes about specific social groups. In a health context, these may be ideas that have been represented in films, heard during the academic training, in conversation with colleagues, or through other health professionals. We stress that these characteristics may or may not reflect your personal beliefs about these groups. Think of Black Africans as a group rather than a specific individual you may know. Please note that we are not interested in your personal beliefs, but those that are shared by health professionals in general”. Then, participants were asked to indicate as many cultural stereotypes about Black Africans’ health behaviors as they could remember. Afterward, participants completed sociodemographic information and were briefly debriefed [39].

#### Analytical procedure

**Fig 1:**
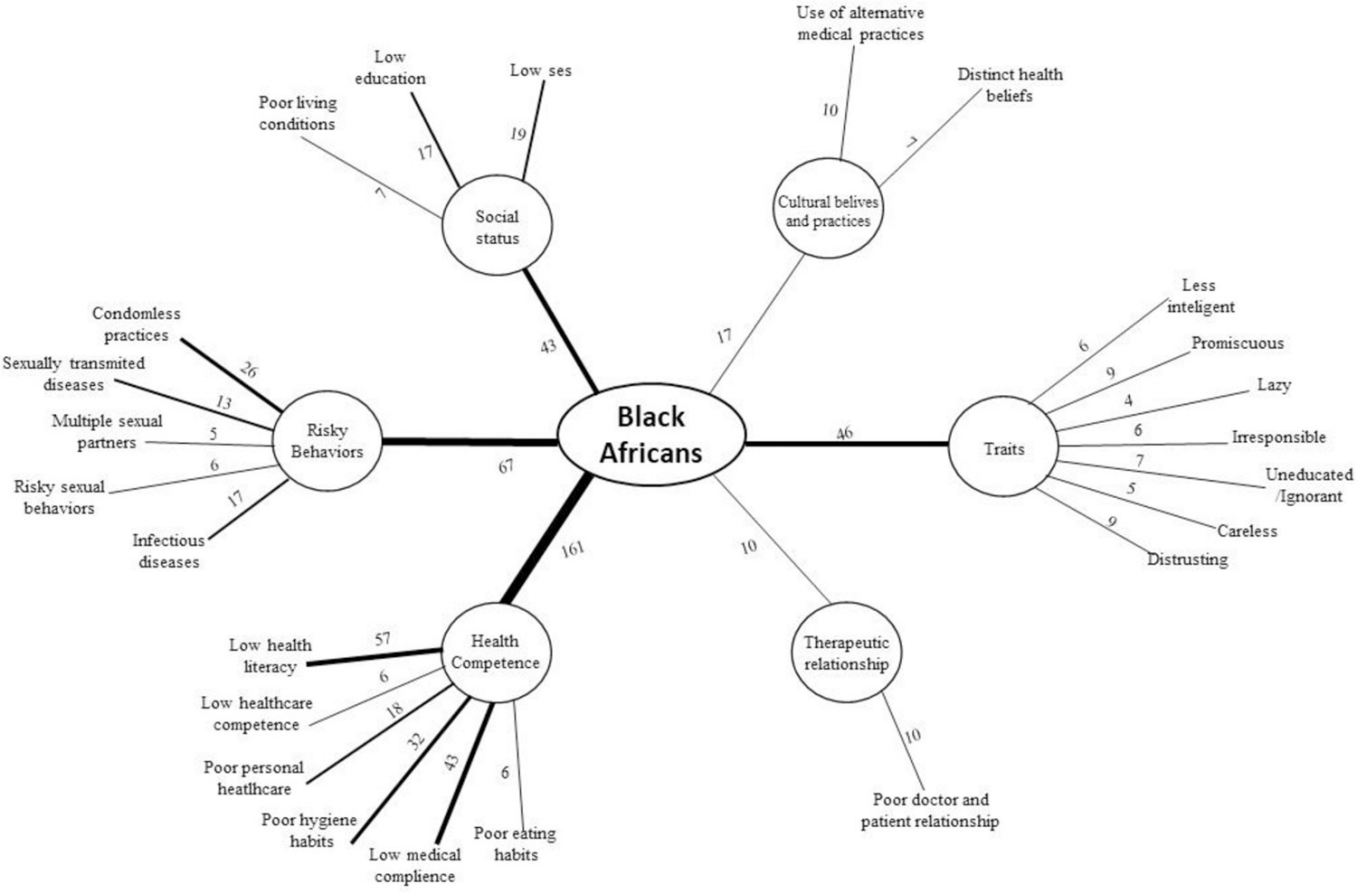
Cultural stereotypes about health behaviors of Black Africans. *Note*. After identifying the units of registration (i.e., text segments that share the same themes) and determining the general categories that emerged from the collected data, the authors created a system to summarize and illustrate the results. The number of units of registration was calculated based on their frequency. Thicker lines in this system correspond only to higher frequencies among categories and registration units.

A content analysis was conducted to explore the content of stereotypes associated with Black people in the health care context [40]. To attain that goal, the content was analyzed by two researchers in three sequential phases—pre-analysis, exploration of the material, and inference and interpretation of results. Any disagreement was discussed and resolved through consensus. In the first phase—pre-analysis—responses collected were organized to systematize the data. This systematization was done in three steps: firstly, participants’ responses were fully read, so-called “floating reading” technique, which allowed to take notes and obtain some impressions of the material; secondly, statements that did not directly reflect health behaviors of Black Africans were removed to keep the focus of the analysis exclusively on inferences about racial stereotypes; and, finally, the elaboration of content indicators was done through text extracts. In the second phase, the development of categories that described the content of stereotypes associated with Black Africans in the healthcare was formed inductively, as they emerged from the data, but also through a theoretical backdrop of the socio-psychological research on racism and health [30,41]. At this stage, the first six categories (i.e., coding system), and then units of registration (i.e. segments of text that share the same themes) were finally created by the consensus of the researchers. Then, the frequency of the units of registration previously identified was quantified. Excel was used to process the data collected. In the last phase, the inferential interpretations of the categories were made. To summarize the relationships found between categories and units of registration, a system was created in Fig 1, in which thicker lines correspond only to higher frequencies among categories and units of registration. In this way, we show in an exploratory way which categories and units of registration were most frequently identified in the collected material. We note that the researchers involved in the phases mentioned had previous experience with the content analysis technique use as proposed by Bardin [40], as well as expertise in the area of prejudice and discrimination against social minorities in healthcare.

### Results

Participants in the study reported that the following aspects are associated with Black Africans: traits, risk behaviors, social status, health competence, patient-provider relationship, and cultural practices (*see* Fig 1). Concerning the traits category, participants in the study reported that Black Africans are identified as promiscuous (9); distrusting (9); less intelligent (6); irresponsible (6); uneducated/ignorant (7); careless (5); and, lazy (4). These traits are illustrated in the following textual extracts:

[Black Africans] *They are irresponsible and do not comply with obligations*, *being little concerned*.

*They are lazy, promiscuous and ignorant*.

Another emerging theme was associated with risk behaviors, where Black Africans were associated with condomless practices (26); infectious diseases (17); sexually transmitted diseases (13); sexual risk behaviors (6); and multiple sexual partners (5).

[Black Africans] *They have less knowledge about HIV infection*, *so they get more infected*.

*They have a higher risk of sexually transmitted infections*. *They have several partners*.

Another category was related to social status, where Black Africans are portrayed as having low socio-economic status (20), a low level of education (15); and poor living conditions (7).

[Black Africans] *They have low education*. *They have few economic resources*. *They have reduced sanitary conditions*. *They have low housing conditions*.

As to the health competence category, participants associate Black Africans with low health literacy (57); low patient compliance (43); poor hygiene habits (32); poor personal healthcare (18); low healthcare competence (6); and poor eating habits (6).

[Black Africans] *They have little health care*, *do not comply with therapy*. *They stink*. *They have poor health education*. *They have less care with their personal hygiene and food*. *They have poor nutrition*.

The last two categories emphasize patient-provider relationship and cultural practices. The former describes the doctor-patient relationship (i.e., Black patient—White provider) as being poor (10), while the latter, shows how the use of alternative medical practices (10) and distinct health beliefs (7) are associated with Blacks.

[Black Africans] *They make it difficult for healthcare workers to explain things to them*.

*They do not believe in physicians*. *They have different beliefs about health, which leads them to be not interested in what the doctor could offer. They have multiple natural treatments, rituals and healers that are sometimes preferred over prescribed drugs*.

## Study 2. The role of patients’ racial stereotypes in medical judgements about patients

Study 2 was designed to investigate how racial stereotypes influence medical judgment for Black Africans and White patients. When activating a stereotype, individuals are reminded of the characteristic attributes of members of a particular group or category [28]. When activating a negative stereotype consistent with a target’s group, the target’s evaluation should result more unfavorably [37,38]. But the magnitude of this disfavor should be different for targets of the in-group (cued as Whites) and out-groups (cued as Blacks), being the latter the most likely target of a greater disparity. In addition, the process of stereotyping may result more unfavorable towards Black patients for participants high in racial prejudice. Earlier evidence showed that Black patients (*vs*. White) were less likely to be recommended for the appropriate medical treatment, particularly among physicians showing high implicit prejudice [25]. Nevertheless, in this study physicians evaluated a Black or a White patient, with the exactly same symptoms. Therefore, the external validity of this finding may be threatened since, in actual medical practice, physicians often see a sequence of patients with similar (albeit not identical) symptoms and medical conditions. Thus, how a patient is fairly treated may be affected by the social comparison situation when evaluating patients from different groups at a time. In the current study, participants evaluated a Black African and a White patient. We hypothesized that the Black patient is more likely to be perceived as less compliant in medical cases involving stereotypically-black behaviors, than those involving non-stereotypic ones. Importantly, this effect is thought to be stronger among individuals high in prejudice, in that stereotypic (*vs*. non-stereotypic) inferences negatively affect more strongly evaluations about the Black (*vs*. White) patient compliance.

### Method

#### Participants

A total of 219 White medical students were invited through a server list to participate in an online experiment, collected through Qualtrics platform, in exchange for a 10€ gift card.

Data from 11 participants (7%) was removed (7 participants because of dual citizenship—Portuguese and other—and 4 for not reaching the survey’s end). The final sample comprised 208 medical students (66% female, Age: *M* = 22.60, *SD* = 2.09) ranging from first to sixth medical school year (*M* = 3.58; *SD* = 1.34). A sensitivity analysis indicates that this sample size has a power of .80 to detect an effect of *f* = .20 or higher with a *p* = .05 as estimated by WebPower [42]. We used a mixed-model design in which the participants were randomly assigned to a 2 (target race: white *vs*. black) × 2 (stereotypicality: stereotypic *vs*. non-stereotypic), and the first factor was manipulated within participants. Racial prejudice was measured and the main dependent variable was the perception of patient compliance.

#### Decision-making task procedure

Participants were told that they would take part in a research study about medical decision-making processes. In the decision-making task, participants read information on hepatitis C and on the rationalization of the HCV treatment. Regarding the rationalization of HCV treatment, participants read the following sentence, “it is important to note that access to HCV treatment is universal, but the cost of treatment (7000 euros per patient) suggests efficient and rational use of the appropriate oral antiviral drugs.” Next, each participant was asked to imagine themselves as a doctor, a member of a panel with the mission of recommending patients for treatment of hepatitis C. Participants viewed three clinical cases of patients. The clinical case vignettes described a male target (i.e., Black; White) who came to the consultation after seeing the primary care physician, where it was confirmed that the patient tested positive after an HCV screening test was performed. Also, it stated that the liver disease was assessed by FibroScan and that it was in grade F3 (advanced fibrosis) on a scale of F0 to F4. The first and the third clinical cases corresponded to the critical trials. The second case was a filler case. Other than that, clinical cases were similar in age and health status (*see* S1–S3 Figs).

Previous studies that used similar decision paradigms have shown that the order of presentation of the target is important because it sets a frame of reference that can lead to differences between groups in medical decision making [*see* Study 1; 1]. The frame of reference refers to which target is evaluated first. If a Black African patient is evaluated *first*, that represents an intergroup comparison, because it activates an intergroup context; if a White patient is evaluated *first*, that represents an intragroup comparison, because it activates an interpersonal context. Because the identity-of-first-group is important, and likely to produce intergroup differences [43] the order of presentation of the cases in this study was not counterbalanced to ensure that both cases were assessed in an intergroup frame of reference—with outgroup patients (referred to as Blacks) being assessed first than ingroup patients (referred to as Whites) [44]. Therefore, the target order was hold constant: White medical students evaluated first the Black target, followed by the White one.

After the decision-making task, participants completed sociodemographic information and were debriefed. Ethical approval was obtained from the Ethics Committee of the Faculty of Medicine of the University of Lisbon.

#### Instruments

*Medical recommendation*. One item measured in a seven-point scale from not at all (1) to very much (7) to what extent would they recommend the patient for the treatment of hepatitis C.

*Patient compliance*. Two items measured in a seven-point scale, from not at all (1) to highly (7) how likely would the patient be to follow doctor recommendations and to responsibly take care of his health. The items were averaged together to form a composite measure (*r* = .91).

*Racial prejudice*. To assess participants’ racial prejudice, we used an adapted version of the Subtle and Flagrant Prejudice Scale [45,46]. This measure is composed by six items evaluating attitudes towards Blacks (e.g., ‘As far as honesty is concerned, I think that White people and Black people are very different’; ‘I would be bothered if a close relative married a Black man of a similar social class’; α = .82) in a seven-point scale, ranging from 1 (I completely disagree) to 7 (I completely agree).

#### Stimuli

*Stereotypicality manipulation*. Participants were randomly assigned to one of the two conditions: participants either see the Black patient associated with (a) unprotected sex practices (stereotypic condition) or with (b) shared tubes for cocaine consumption (non- stereotypic condition).

*Race prime*. On the top of each clinical vignette, a photograph of a fictitious patient was added as a way to highlight the patient’s racial category. Face images from the Face Research Lab London Set project [47] were blurred in order to exclude effects of facial features stereotypicality (i.e., Afrocentric features, such as darker skin tone or wider nose).

### Results

#### Medical recommendation

A 2 (Patient Race: Black *vs*. White) × 2 (Stereotypicality: stereotypic *vs*. non-stereotypic) mixed-design ANOVA with repeated measures on the first factor yielded a significant effect of Patient Race *F* (1, 172) = 64.24, *p* = .001, *η*_*p*_^*2*^ = .27, and a significant effect of Stereotypicality was found, *F* (1, 172) = 5.30, *p* = .02, *η*_*p*_^*2*^ = .03. The results show that the Black patient (*M* = 5.99, *SE* = .09) received a higher prioritization than the White patient (*M* = 5.34, *SE* = .09). Moreover, medical recommendation was higher in the stereotypic (*M* = 5.84, *SE* = .11) relative to the non-stereotypic condition (*M* = 5.49, *SE* = .11). The patient race effect was qualified by the stereotypicality of infection, *F*_*patient race× stereotypicality*_ (1, 172) = 49.87, *p* = .001, *η*_*p*_^*2*^ = .23. Follow up pairwise comparisons show that in the non-stereotypic condition the Black patient (*vs*. White) is more likely to be recommended, *F* (1, 172) = 108.65, *p* < .001, *η*_*p*_^*2*^ = .39. In the stereotypic condition, the Black and White patients are equally likely to be recommended, *F* (1,172) = .47, *p* = .49, *η*_*p*_^*2*^ = .00 (*see* Table 1).

**Table 1:** Means and standard deviation of responses to medical recommendation and patient compliance measures.

	Stereotypicality							
	Medical Recommendation				Patient Compliance			
	Stereotypic		Non-stereotypic		Stereotypic		Non-stereotypic	
	*M*	*SD*	*M*	*SD*	*M*	*SD*	*M*	*SD*
Black	5.88_a_	1.21	6.10_a_	1.06	4.46_c_	1.26	5.30_d_	.94
White	5.80_a_	1.15	4.88_b_	1.14	4.56_c_	1.16	4.37_c_	1.16

*Note*. Means with different subscripts in the same row or column are significantly different at *p* < .05. *M*—Mean; *SD—*Standardized Deviation.

#### Patient compliance

Again, for patient compliance, a 2 (Patient Race: Black *vs*. White) × 2 (Stereotypicality: stereotypic *vs*. non-stereotypic) mixed-design ANOVA with repeated measures on the first factor yielded a significant effect of Patient Race, *F* (1, 172) = 24.95, *p* = .001, *η*_*p*_^*2*^ = 12, and a significant effect of Stereotypicality was found, *F* (1, 172) = 4.56, *p* = .03, *η*_*p*_^*2*^ = .03. The results show that the Black patient (*M* = 4.88, *SE* = .09) is perceived as more compliant than the White patient (*M* = 4.46, *SE* = .09). The effect of Stereotypicality on patient compliance differed from that described above, that is, patient compliance was higher in the non-stereotypic (*M* = 4.83, *SE* = .11) compared with the stereotypic condition (*M* = 4.51, *SE* = .10). Again, the effect of patient race on perceived compliance was qualified by the Stereotypicality of the infection, *F*_*patient race× stereotypicality*_ (1, 172) = 37.64, *p* = .001, *η*_*p*_^*2*^ = .18. Follow up pairwise comparisons showed that the effect of Stereotypicality on patient compliance was significantly different for Black patients, *F* (1, 172) = 24.34, *p* < .001, *η*_*p*_^*2*^ = .12, but not for White patients, *F* (1, 172) = 1.20, *p* = .28, *η*_*p*_^*2*^ = .007. Thus, whether the source of infection is stereotypic or non-stereotypic affects how likely the Black patient is perceived to follow doctor recommendations and to take care of his health. As hypothesized, participants evaluated the Black patient, with a source of infection implying black stereotypic behavior as less compliant (*M* = 4.46; *SE* = .12) compared with the Black patient with a source of infection implying non-black stereotypic behavior (*M* = 5.30; *SE* = .12). White patients were assessed in a similar manner, regardless of the stereotypicality of the source of infection.

Next, we investigated whether Black patients will be seen as less compliant than white patients, when engaging in stereotypical behaviors, especially among individuals high in prejudice. We used a linear mixed model specific equivalent to a random-effects analysis of covariance, where the patient race is the factor and the higher level explanatory variables are the covariates. Statistically, each participant is a dyad, composed of two scores–a score for the Black target and a score for the White target. Thus, each dyad has two scores on Y. The level-1 predictor variable is the target race, the level-2 predictor variable is the participant; the level-3 predictor variable is the experimental condition, the source of contagious (stereotypic vs. non-stereotypic). Because there are only two observations at level 1, there is not much variation on the slopes from participant to participant. Thus, coefficients from the first stage of analysis must be constrained to be equal across all participants [48]. In other words, the model will contain all the fixed effects for the predictors and interactions and two random effects (variation in the intercepts and error variance) across model estimation. This does not bias the results, because variation is properly included in the error variance [48]. As predicted, we found a three-way interaction for patient compliance, *B* = .42, *SE* = .19, *t* = 2.12, *p* = .04 (*see* Table 2). In the next step, we broke down the three way-interaction. We examine the effect of the patient race, moderated by the stereotypicality, at each level of racial prejudice. Since the Patient race × Stereotypicality interaction was significant, *B* = 1.05, *SE* = .17, *t* = 6.37, *p* = .001, we examined to which degree race-based distinctions in priority would vary as a function of stereotypicality for low prejudiced and for high prejudiced participants. Thus, we calculated the simple slopes for the effect of stereotypicality at one standard deviation below the mean and at one standard deviation above the mean of racial prejudice.

**Table 2:** Summary of multilevel analysis for variables predicting the perceived patient compliance.

Predictor	Patient Compliance		
	*B*	*SE (B)*	*t*
Patient race: Black	4.470	.117	38.047^***^
Patient race: White	4.571	.117	38.912^***^
Stereotypicality	-.218	.170	-1.281
Racial Prejudice	-.205	.129	-1.586
Patient race × Stereotypicality	1.051	.165	**6.375** ^***^
Patient race × Racial Prejudice	-.051	.125	-.410
Stereotypicality × Racial Prejudice	-.032	.205	-.156
Patient race × Stereotypicality × Racial Prejudice	.420	.200	**2.118** ^*^

Note.

*p < .05

***p < .001. For stereotypicality, 0 = Non-stereotypic, 1 = Stereotypic.

**Fig 2:**
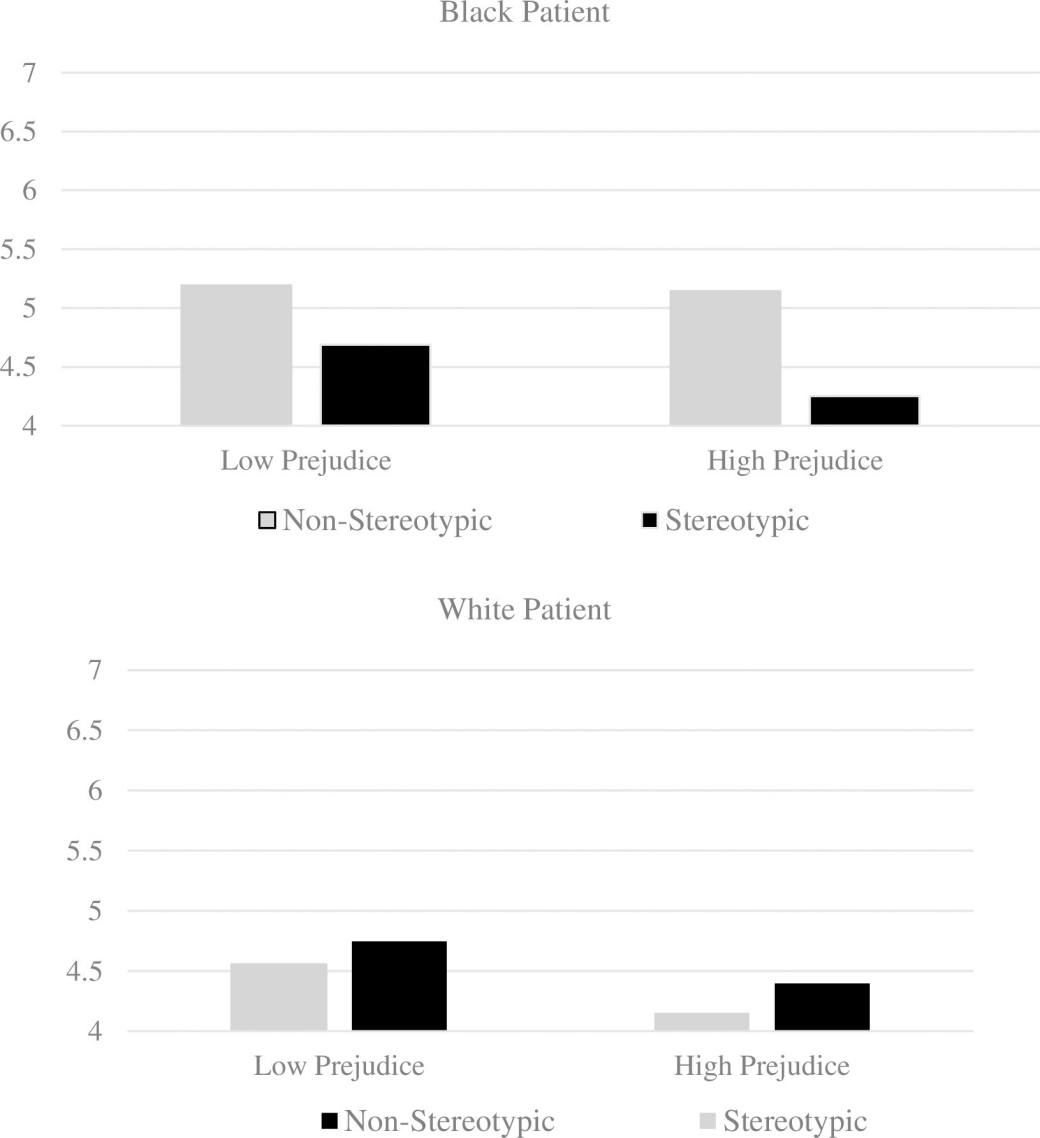
Predicting perceived compliance for Black and White patients as a function of stereotypicality and racial prejudice.

The results showed that the effect of race on patient perceived compliance does indeed vary according to the level of prejudice, but only for the Black patient. When the behavior is stereotypically Black, the Black patient is perceived less favorably compared to the non-stereotypic behavior, both for participants low and high on racial prejudice. However, the magnitude of this difference is higher among those participants high on racial prejudice (*see* Fig 2). Moreover, when the behavior is stereotypic, participants high on racial prejudice tend to perceive the Black patient as less compliant (*M* = 4.25, *SE* = .16) compared to participants low on racial prejudice (*M* = 4.67, *SE* = .17). However, when the behavior is non-stereotypic, the Black patient is perceived to be equally compliant for those with high prejudice (*M* = 5.41, *SE* = 19) and low prejudice (*M* = 5.19, *SE* = .18). For the White patient, we found no significant differences.

## Discussion

We explored the stereotypes associated with Black people in the health care context and to what extent the relationship between patient race and medical judgment regarding HCV-treatment changes as a function of the stereotypical information and the level of racial prejudice.

Previous research has documented the relationship between race and recommendation for HCV-treatment [4,10]. The current study goes beyond the association between race and medical judgment. We introduce two relevant psychosocial mechanisms–stereotypes and racial prejudice–to provide a possible explanation for that association. In addition, we explain how stereotypical consistent information may unintentionally affect medical judgements about Black patients, but not about White patients. To that end, in Study 1 we assessed explicit negative stereotypes about Black patients. Then in Study 2, we experimentally manipulated (a) a Black-stereotypic and (b) a non-Black-stereotypic information portrayed in a clinical case, to examine to what extent the relationship between race and perception of patient compliance in hepatitis C treatment is affected by stereotypic cues and heightened by racial prejudice.

Using a theme-based categories analysis, results from Study 1 offered relevant dimensions of racial stereotypes in the health care context. In general, the six dimensions that emerged from the analyses show that the content of racial stereotypes in health are predominantly unfavorable. Described in a negative way, stereotypes that Black Africans are seen as lazy, irresponsible and unintelligent have been reported in previous work [25,26,32,33,49]. The content of these stereotypes is related to the perception of the social structure, where Blacks are seen as a low status group associated with poor living conditions, low levels of education, and a lower socioeconomic status [50].

Also of importance are the references to an unhealthy lifestyle (poor eating habits; poor personal hygiene) and medical care, as well as references to health illiteracy reported by participants. These stereotypical associations between race and a certain inability to manage a healthy lifestyle and compliance are important elements in the quality of the doctor-patient relationship also found in other studies [30].

The reference to the difficulties in the doctor-patient relationship and the unfamiliarity of professionals to cultural and religious practices had already been identified in other studies, showing that interpersonal relations with Black Africans patients are viewed as more difficult. And one of the explanations rely on the fact that such patients hold different beliefs about health, making it more difficult for physicians to establish a good interracial doctor-patient relationship [41].

Another important aspect is the reported association between Black Africans and health risk practices. Previous work had shown an association between this group and risky sexual practices within HIV-domain [35]. These findings extend this stereotypical association to other infectious diseases, such as hepatitis C. Importantly, contrary to other research, the use of alcohol or other drugs did not emerge as being, at least at an explicit level, stereotypical of this group [34].

Based on these last findings, in Study 2 we experimentally manipulated stereotypic content, and tested the hypothesis that when a Black patient is described with a stereotypical cue, they are more likely to be perceived as less compliant, than when described in a non-stereotypic fashion. In addition, this predicted effect is thought to be stronger among individuals high in prejudice. The results generally supported the hypotheses, both confirmed through pairwise comparisons and in the three-way predicted interaction. The impact of stereotypicality on patient compliance shows that a Black patient is seen as less compliant when displaying a stereotypically-Black behavior (i.e., as explanation for the contagion), even though this behavior is unrelated with the issue of compliance (an aspect previously unaddressed). And this impact of stereotypicality on patient compliance varies as a function of racial prejudice, but only for Black patients.

Overall, our findings contribute to the literature on racial stereotyping in healthcare by showing the impact of explicit negative stereotypes on the evaluation of patients in need for medical treatment [27,31,35], suggesting that when the information provided about a Black patient is consistent with a stereotype about Blacks, the evaluation of the Black patient tends to be more ambivalent. In addition, these findings indicate that racial prejudice magnifies the strength of such an effect. There is evidence that providers as a group often hold explicit negative stereotypes about racial /ethnic/minority patients (e.g. unintelligent, noncompliant, sexually promiscuous), and that these stereotypes may be associated with preferences for treatment recommendations [30,35,51]. To our knowledge, our experiment is the first that experimentally manipulated stereotypicality in the context of medical judgement and provides further understanding about medical students’ vulnerability to stereotyping (in a different national context and pertaining to a different disease and treatment).

Regarding hepatitis C treatment recommendation, the findings also show an effect of race as recommendation for treatment was found to be higher in Black than White patients. Yet, this effect is driven primarily by the White patient in the non-stereotypic condition. In other words, when the White patient is primed with the non-stereotypic condition, they are significantly more punished compared to the other experimental conditions. Thus, both stereotypic and non-stereotypic conditions appear to activate different heuristics for White and Black targets. One possible explanation is that the causal inferences used to compare Black and White patients were different, and therefore had led to different judgments of deservingness [52]. While a White patient having a past of drug use, can be seen as an attribution of higher internal responsibility, and therefore a higher punishment, because this target has greater intellectual and social resources and greater control over their life. The same situation in a Black patient target may have triggered inferences of greater external responsibility, due to a lack of personal and social resources, which reduces the guilt of the target and may have evoked a paternalistic reaction of high warmth and low competence. Indeed, the Black patient was treated more favorably in the prioritization for the medical treatment, yet was perceived substantially less competent than the White patient. This pattern could be explained by an underlying concern with social desirability or could also be construed as a compensation effect, materialized in an outgroup favoritism [53]. The compensation effect hypothesis proposes that, when people evaluate their own and another group, the search for positive differentiation drives a favoritism toward the outgroup, on one of the two fundamental dimensions of social perception, competence and warmth [53,54]. In fact, although the Black patient was more favored in the prioritization of treatment, they are perceived as less competent. One possible interpretation is that the Black patient may have been perceived as warmer, but as less competent, invoking in the participants a paternalistic reaction, expressed in a desire to favor the Black patient and to punish the White patient in the treatment prioritization. Future studies should address what is accounting for the recommendation of the White patient.

Looking at the perception of patient compliance, our assumption was that when the Black patient appeared associated with a stereotypic cue, a less favorable evaluation would occur. And in contrast, when associated with a non-stereotypic cue, a more positive evaluation would take place. Our findings support this assumption taking into account the unpredicted compensation effect. Overall, there was a compensation effect where the Black patient was more likely to be recommended and perceived as competent. Nonetheless, the findings also showed how this effect substantially decreased by the presence of stereotypical consistent information, showing likewise how the impact of stereotypes produced a more unfavorable evaluation toward the Black target. Thus, the results show that the assumption remains in line with other studies regarding the racial stereotypes consistency hypothesis [38]. Medical students behave in a less favorable way when making a decision that is consistent with the stereotypes of Blacks (e.g., risky sexual behaviors) and Whites (e.g. recreational drug use) and react in a more favorable way in making a decision that is inconsistent with the stereotypes of Blacks. In this study, the stereotypical content manipulated was unprotected sexual practice, following Study 1’s results but also a behavior which had already been shown to be a mechanism by which medical students discriminated more against Black than White people when prescribing PrEP [35]. This finding is important because it identifies a content of racial stereotyping in health with emphasis on at least two areas of medicine, often associated with marginalized communities—HIV and now, hepatitis C.

Importantly, our most innovative finding in the current research is how medical students’ racial prejudice interacts with stereotypical cues that are activated spontaneously and effortlessly during medical decision-making processes in the context of hepatitis C treatment. Our results showed that provider’s racial prejudice enhanced the impact of stereotyping on the perception of patient compliance, only when the patient was portrayed as Black. Specifically, the Black patient was perceived as less compliant when the source of their disease was consistent with group-stereotypes, in participants high on racial prejudice (*vs*. participants low on racial prejudice). For the White patient, no differences were found. Thus, the impact of a group-stereotype inferred from a disease led to a less favorable outcome for Black patients, especially among providers high on racial prejudice. These findings are important because they show that group-based stereotypes affect critical dimensions of medical evaluation, such as medical compliance. Despite the presence of racial prejudice (mostly at an implicit level) among medical students, there are very few studies that show an association between implicit racial prejudice and negative healthcare outcomes [55]. The current research contributes to this literature by showing that racial prejudice moderates the impact of racial stereotypes on judgements about patient compliance, which advances the ways in which these biases contribute to healthcare inequalities.

Future work with medical students should consider further training where the future doctors are to be made aware of the existence of these preconceived notions about different racial groups and how these may affect their judgment towards members of these groups. Incorporating the study of these (hidden) factors into medical education may help to develop innovative strategies to reduce physicians’ bias and its contribution to racial inequalities in health care.

## Limitations and future research directions

To further our understanding of how stereotyping affects both perception of patient compliance and treatment decisions—particularly those decisions that have a great impact on people’s lives, such as hepatitis C treatment decisions—more research is warranted. The current research only focused on the impact of racial stereotypes associated with Black people. We do not know yet how stereotypical cues associated with other social groups (e.g., Whites) would impact medical students’ reactions and decisions towards the targeted group. Thus, it would be highly beneficial for research such as this to evaluate how stereotypicality of Black and White people are the main causes of contagion of hepatitis C, and test its impact on treatment decisions.

The evidence supporting a compensation effect on treatment recommendations and patient compliance is not surprising. In studies using clinical vignettes, participants are likely aware about the racial intent of the experiment, and therefore may engage in a motivated and deliberate self-regulatory behavior [56]. Especially because the antiracism norm supporting nondiscriminatory medical practice emphasizes that good healthcare providers are egalitarian and treat patients fairly [57,58]. One possible way to overcome this limitation is using a less obvious manipulation of patient race. For example, in a previous study, physicians were subliminally primed with words related to Black, Latinos or White [59]. The assumption was that subliminal priming would activate the categorical dimension (i.e., patient race). Then participants were asked to read a vignette about a 62-year-old female patient who complained about chest pain. The results show that using a subliminal race prime predicted the effect of race on treatment recommendations. Moreover, there is evidence that time pressure or cognitive load increases the likelihood of stereotype usage [37]. Providers may be especially vulnerable to the use of stereotypes in forming impressions of patients since time pressure and the need to manage very complex cognitive tasks are frequent in their work [30]. Thus, it would be interesting for future work to use an implicit race prime, combined with a time pressure manipulation during the decision-making task.

The results presented here resulted exclusively from explicit measures. Yet, recent theorizing in the field of health inequalities has drawn attention to the interactive use of implicit and explicit measures on racial/ethnic healthcare bias. One of the reasons is grounded on the Aversive Racism framework [57,60]. Aversive racism is a specific type of contemporary bias held by people who (a) endorse egalitarian values and beliefs, (b) believe themselves to be unprejudiced, but (c) unconsciously hold negative beliefs about Blacks and other out-groups, and (d) subtly discriminate in ways that are ambiguous and indirect and that can be rationalized as something other than racial discrimination [61]. Results from three studies have shown that providers profiled as aversive racists (i.e., those who score high on an implicit measure of prejudice and low on an explicit measure of prejudice) were rated less positively by their Black patients than any other primary care providers and displayed less engagement, more negative affect, and less positive affect than other providers, particularly when they interacted with Black patients who reported any incidence of prior discrimination [18,58,62]. Future work could address such issue by using a combination of scores between implicit and explicit measures [63]. Despite the limitations identified, this work takes a step forward by experimentally manipulating racial stereotypes and analyzing their impact on the cognitions and behavioral intentions of future health professionals. In doing so, it contributes with empirical evidence to the effect of stereotyping on inequalities in health care.

## Supporting information

S1 Fig Example of the clinical case vignette of the Black patient. (TIF)

S2 Fig Example of the clinical case vignette of filler case. (TIF)

S3 Fig Example of the clinical case vignette of the White patient. (TIF)
