# Supplementary figures and images for: The role of stereotypical information on medical judgements for black and white patients
Source: PLoS One. 2022 Jun 8;17(6):e0268888. doi: 10.1371/journal.pone.0268888 (PMC9176779; doi:10.1371/journal.pone.0268888)

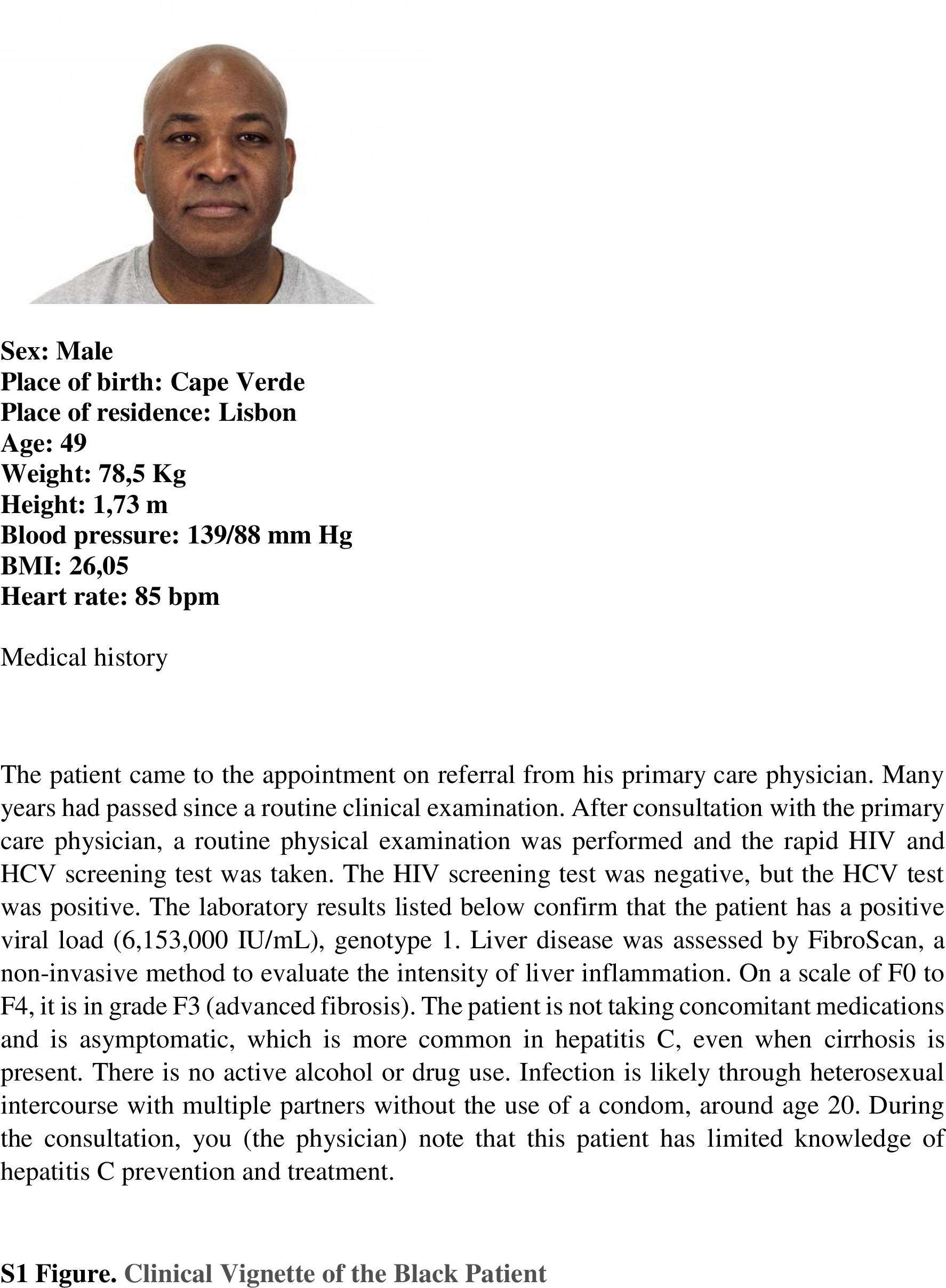

Supplement: S1 Fig — (TIF) [file pone.0268888.s001.tif]

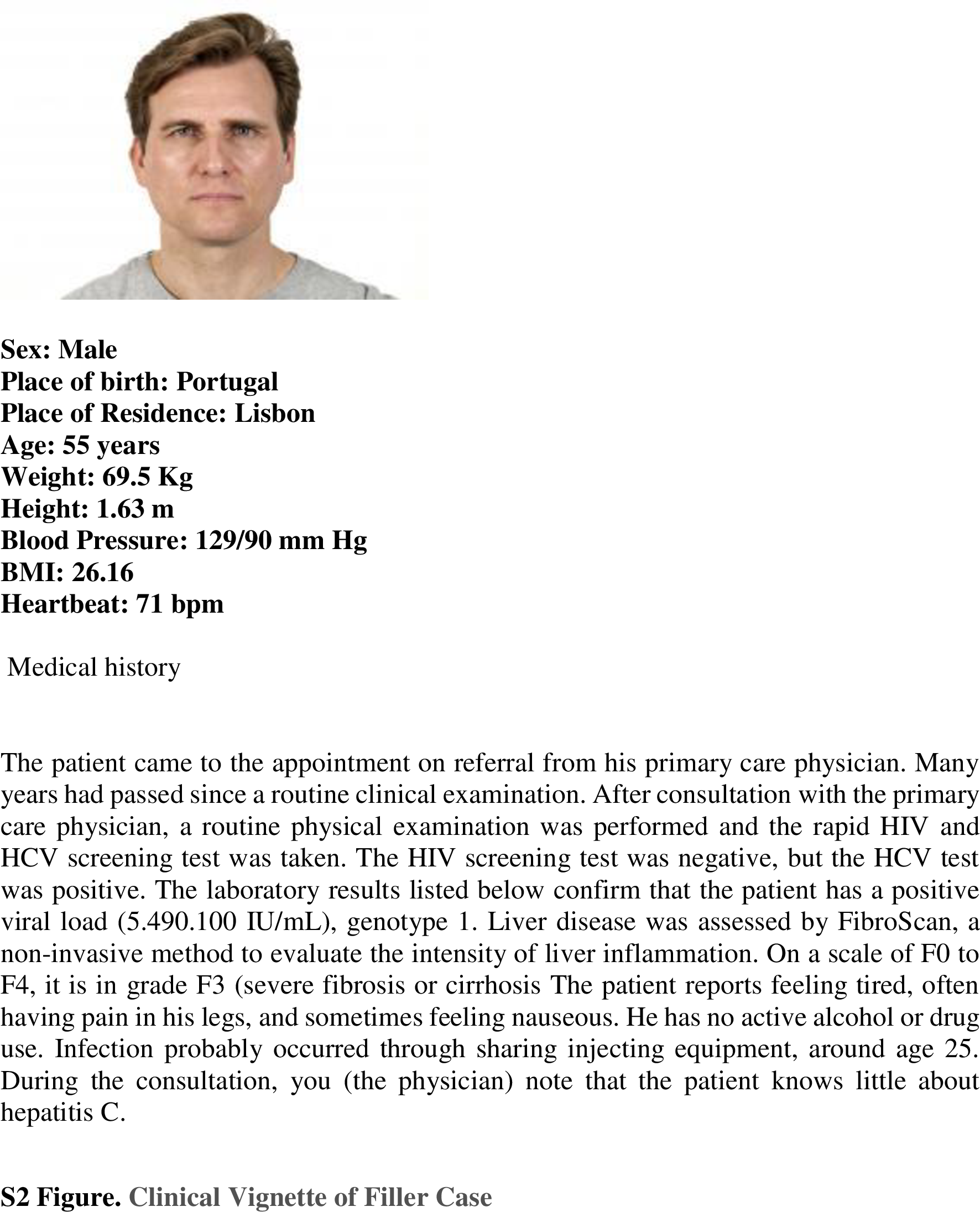

Supplement: S2 Fig — (TIF) [file pone.0268888.s002.tif]

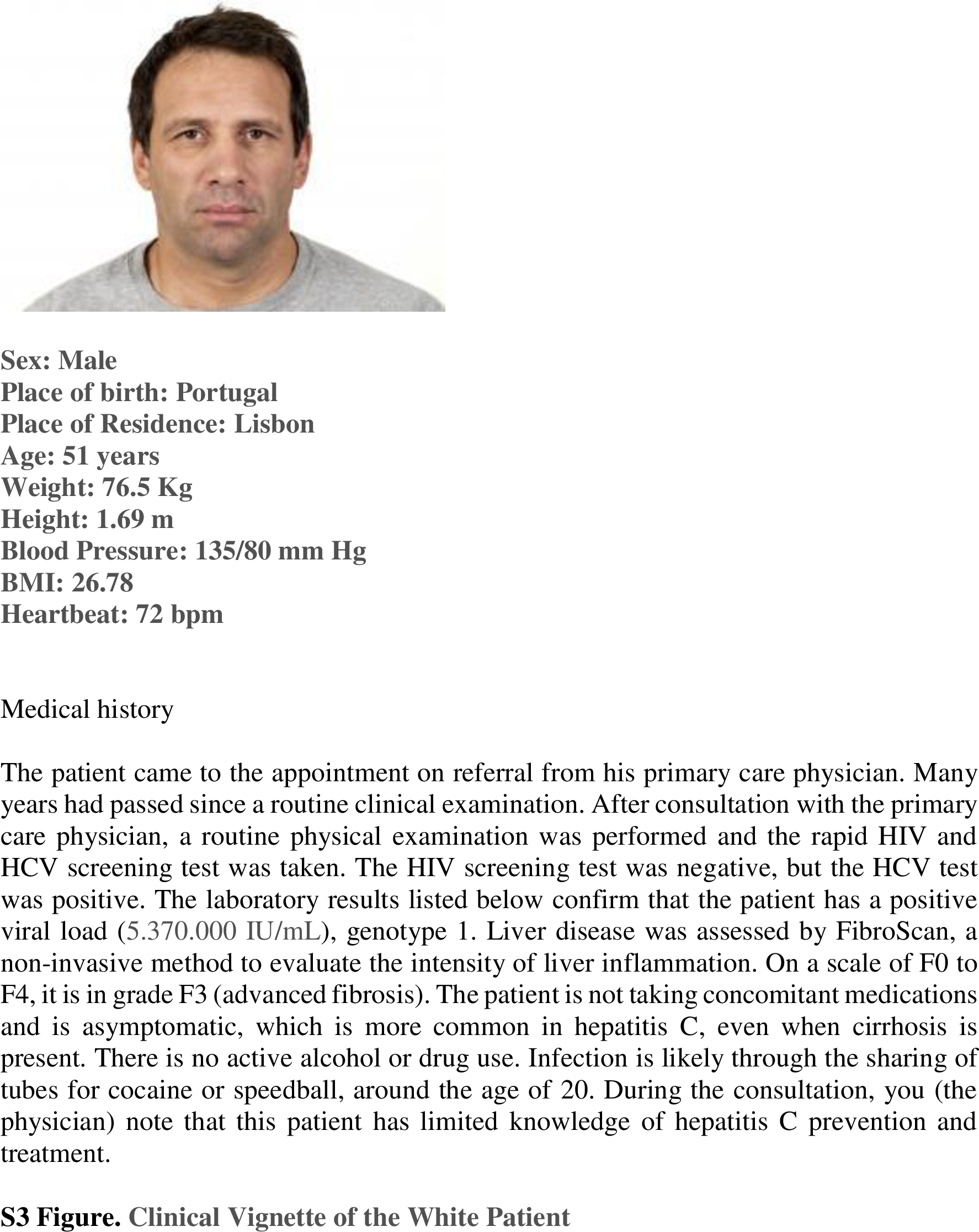

Supplement: S3 Fig — (TIF) [file pone.0268888.s003.tif]
